# Spin–orbit coupling of light in asymmetric microcavities

**DOI:** 10.1038/ncomms10983

**Published:** 2016-03-18

**Authors:** L. B. Ma, S. L. Li, V. M. Fomin, M. Hentschel, J. B. Götte, Y. Yin, M. R. Jorgensen, O. G. Schmidt

**Affiliations:** 1 Institute for Integrative Nanosciences, IFW Dresden, Helmholtzstrasse 20, D-01069 Dresden, Germany; 2 Institut für Physik, Technische Universität Ilmenau, Weimarer Straße 25, D-98693 Ilmenau, Germany; 3 Max Planck Institute for the Physics of Complex Systems, Nöthnitzer Straße 38, 01187 Dresden, Germany; 4 Material Systems for Nanoelectronics, Chemnitz University of Technology, Reichenhainer Straße 70, 09107 Chemnitz, Germany

## Abstract

When spinning particles, such as electrons and photons, undergo spin–orbit coupling, they can acquire an extra phase in addition to the well-known dynamical phase. This extra phase is called the geometric phase (also known as the Berry phase), which plays an important role in a startling variety of physical contexts such as in photonics, condensed matter, high-energy and space physics. The geometric phase was originally discussed for a cyclically evolving physical system with an Abelian evolution, and was later generalized to non-cyclic and non-Abelian cases, which are the most interesting fundamental subjects in this area and indicate promising applications in various fields. Here, we enable optical spin–orbit coupling in asymmetric microcavities and experimentally observe a non-cyclic optical geometric phase acquired in a non-Abelian evolution. Our work is relevant to fundamental studies and implies promising applications by manipulating photons in on-chip quantum devices.

## Abstract (web summary)

Optical spin–orbit coupling is known to occur in open systems such as helical waveguides. Here, the authors enable spin–orbit coupling of light confined to a closed path within an asymmetric optical microcavity and demonstrate a non-cyclic Berry phase acquired in a non-Abelian evolution.

## Graphical abstract

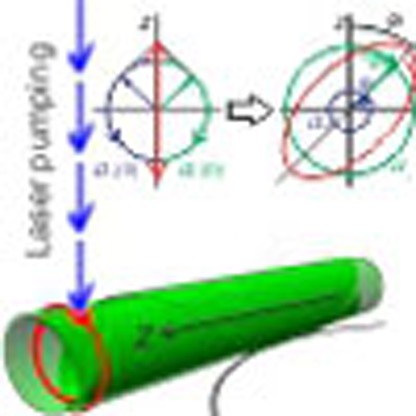

In optics, spin–orbit coupling leads to two important observable effects, the geometric phase^1^,^2^,^3^,^4^,^5^,^6^ and the spin Hall effect^3^,^6^,^7^,^8^, which play an important role in a surprisingly large number of physical contexts^7^,^9^,^10^,^11^,^12^. The geometric phase has been generalized from a cyclic and Abelian context to non-cyclic and non-Abelian cases^13^,^14^,^15^, which have been realized in many physical systems, such as spinning neutrons^16^ and superconducting artificial atoms^17^. In optics, it is of fundamental interest to realize a non-cyclic geometric phase acquired in a non-Abelian evolution by enabling optical spin–orbit coupling in a weakly anisotropic medium^3^,^18^. Optical spin–orbit coupling has been observed in open systems by the refraction across an interface^7^ or in the propagation along helical waveguides^3^,^19^.

Here we report on the spin–orbit coupling of light confined to a closed path within an asymmetric optical microcavity. The polarization state of light is found to change in both orientation and eccentricity due to the occurrence of a geometric phase together with a mode conversion, generating a non-cyclic geometric phase in a non-Abelian evolution.

## Results

### Theory of light evolution applied to optical microcavities

Optical microcavities, which confine light to small volumes by resonant circulation in a dielectric medium, play an indispensable role in a wide range of applications and fundamental studies^20^. In a general theory describing the evolution of light in a dielectric medium, a quantum mechanical diagonalization procedure was applied to the Maxwell equations and Berry's phase theory^18^, where the effective Hamiltonian takes the form:


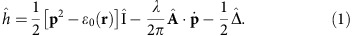


Here **p** is the momentum operator, *ɛ*_0_(**r**) represents the scalar isotropic component of the propagation medium, 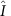 stands for a unit matrix, the matrix 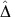 denotes the anisotropic component of the dielectric permittivity, *λ* is the wavelength, 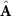 represents the gauge potential, and 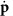 is the derivative of **p**. The Hamiltonian can be divided into three parts 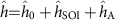. The first part 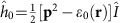 characterizes the ordinary light propagation and interference. The second part 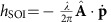 denotes the spin–orbit coupling of photons, and the third part 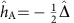 describes the medium anisotropy^18^.

**Figure 1:**
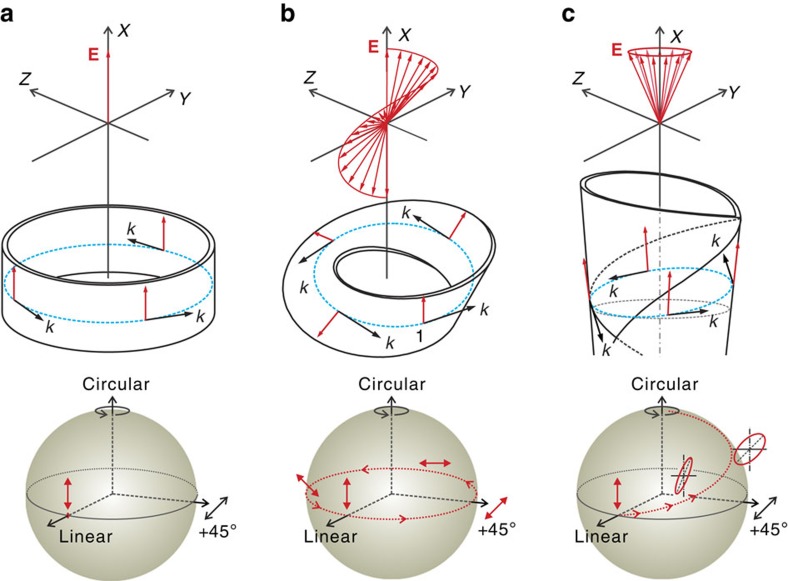
Optical spin–orbit coupling in WGM microcavities (top panel) and the corresponding polarization evolution on the Poincaré sphere (bottom panel). (**a**) In-plane polarized light does not provide orbital angular momentum in a symmetric ring resonator due to the unchanged electric field (**E**) vector with respect to the wave vector **k**, which results in a stationary point on the Poincaré sphere. (**b**) In a Möbius-ring resonator, the twisted electric field **E** along the Möbius strip causes a varying orbital angular momentum for spin–orbit coupling, which results in a cyclic evolution on the Poincaré sphere. (**c**) An effective orbital angular momentum along *X* is generated due to the rotation of the major axis of the electric field **E** regulated by the cone-shaped tube wall of an anisotropic medium, allowing for an interaction with the spin angular momentum, which results in a non-cyclic evolution on the Poincaré sphere. The variations of the major polarization axis of the field **E** (red arrows) are shown with respect to the laboratory coordinate frame (*XYZ*). The blue dashed lines represent light trajectories, while the red dotted lines represent the polarization evolution trace on Poincaré sphere.

In conventional optical whispering-gallery-mode (WGM) microcavities, such as a cylindrical ring resonator (see Fig. 1a), the electric field vector does not change with respect to the wave vector **k**. In addition, the resonant light propagates along a closed-loop trajectory, which is distinct from the open helical trajectories that have been widely used to enable the optical spin–orbit interactions^2^,^3^,^19^,^21^. Unlike the propagation via helical trajectories, the wave vector **k** experiences a trivial evolution when propagating along a closed loop. As such, the optical spin–orbit interaction is irrelevant and the corresponding Hamiltonian contains only the 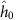 part, which results in ordinary discrete eigenmodes in optical WGM resonators. Experimentally, the eigenmodes manifest themselves by discrete peaks in the resonant spectra. Each peak in the resonant spectrum is formed by self-interference with an integer number of waves along the closed-loop trajectory^20^,^22^. In these systems the optical polarization states are conserved at each resonance.

However, optical spin–orbit coupling can be induced in specially designed cavity structures. For example, one can introduce topology into a WGM cavity by employing a Möbius strip^23^ as an optical micro-ring cavity. Although the wave vector **k** experiences a trivial evolution in this geometry, the transverse electric field twists around during the propagation in the strip (see Fig. 1b). In this way, an effective orbital angular momentum (OAM), similar to that of an optical vortex^24^,^25^,^26^ or transformed light beam^27^, is generated for the spin–orbit coupling. Thus, the effective Hamiltonian takes the form 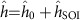, where the spin–orbit coupling leads to the occurrence of a geometric phase. This extra phase leads to a non-integer number of waves for constructive interferences along a closed-loop trajectory, which has been revealed in classical Möbius-ring resonators^28^. Similar to the previously reported helical waveguides^3^,^29^, this behavior represents an Abelian evolution, where the polarization orientation varies, while the polarization eccentricity does not.

Here, we experimentally realize light evolution in the presence of both the spin–orbit interaction and the medium anisotropy in an on-chip cone-shaped microtube resonator. The cone-shaped resonator (see Supplementary Figs 1 and 2) is an asymmetric tube made of a rolled-up SiO_*x*_ thin film^30^ as schematically shown in Fig. 1c. The tube is around 7 μm in diameter with a wall thickness of ∼100 nm. In the microtube cavity, optical WGM-type resonances are established via optical self-interferences along a closed-loop trajectory guided by the cylindrical tube wall. To pump the resonances, a linearly polarized laser (at 532 nm) is focused on the larger diameter tube end, where resonant modes of higher quality (*Q*) factor exist (see Supplementary Fig. 2). The laser excites luminescent defects^31^ in the amorphous silicon oxide microtube, which emit light in the visible spectral range at room temperature. Due to the subwavelength-thin tube wall, photons linearly polarized along the tube wall are allowed to circulate around a closed trajectory within the microtubes^22^, which ensures that the initial state of the resonant light is linearly polarized with the polarization orientated around the tube axis. The photons circulating along the closed trajectory eventually escape from the microtube cavity and can then be measured and analyzed.

When the light propagates in the thin-walled microtube, the electric field vector rotates around the tube axis due to the cone-shape of the microtube (see Fig. 1c). This rotation generates an effective OAM along the tube axis^15^. In conventional WGM cylindrical cavities, the wave vector **k** (indicating the direction of the spin angular momentum ) of the resonant light is orthogonal to the tube axis; thus, there is no possibility to generate the spin–orbit interactions even if there is an OAM along the axis. However, at the larger-diameter-end part of a cone-shaped tube, the average refractive index is made to vary along the tube axis owing to the variation in the number of windings^30^. In this particular geometry, the resonant trajectory slightly tilts out of plane (see Fig. 1c) to reduce the optical path according to Fermat's principle (see Supplementary Note 3). It is this tilted trajectory which causes the spin angular momentum to be not orthogonal to the OAM and which, in turn, enables the coupling between spin and orbital degree of freedom 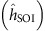. In addition, the resonant light experiences an anisotropic refractive index (see Supplementary Fig. 5) in the asymmetric tube when it propagates along a tilted trajectory, which contributes to the 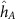 term.

The terms 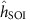 and 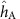 determine the polarization evolution of the optical wave. By expanding the two terms in equation (1) in the basis of Pauli matrices 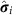 (*i*=1,2,3), the expression 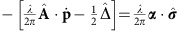 exhibits a similar form to that of electrons under the interaction between spin and orbital magnetic moments, where the vector **α** plays the role of an ‘effective magnetic field'^18^ and 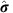 is a vector formed of the Pauli matrices. Based on the Schrödinger equation, the polarization evolution equation reads^13^,^18^


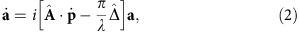


where the polarization state 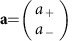 is comprised of right 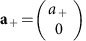 and left 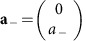 components in the circular polarization basis. A well-known solution of equation (2) takes the form^13^,^18^


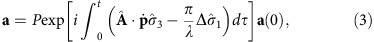


where *P* represents the path-ordering operator and 
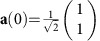
 is the linear polarization state parallel to the tube axis, which is the initial state in this work. The first term in the integral accounts for the Berry phase^1^,^18^


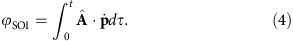


The second term in the integral in equation (3) results in a factor *C*_A_ that originates from the anisotropy of the system. *C*_A_ enables the interplay between the two polarization states that gives rise to the mutual conversion of the right and left circular polarization components **a**_+_ and **a**_−_. One should note that the tensor 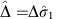 is non-diagonal due to the anisotropy of the medium. In our work the Berry phase is non-cyclic; in general it takes the form^15^


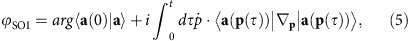


where **a** (**a**≡**a**(*t*)) is the final state after an evolution on an open path in the parameter space. Unlike for the cyclic case, a non-cyclic geometric phase usually cannot easily be derived from equation (5), and practical measurements could be more complicated^32^,^33^,^34^. In the present work, we show a different convenient strategy to measure this noncyclic geometric phase.

Starting from equation (3), one can present the final polarization state in terms of the Jones vector^3^ (see Supplementary Note 6), where the time variable is omitted as we have only access to the measured polarization state at the end of its evolution,


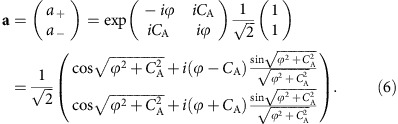


The 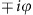 terms denote the geometric phase acquired for each circular basis state. 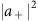 and 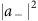 represent the redistributed circular components after the mode conversion, where (see Supplementary Note 6)


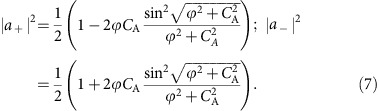


It is the non-diagonal element *iC*_A_ in the matrix in equation (6) that leads to the coupling and, consequently, to a mutual conversion between the two circular polarization components **a**_+_ and **a**_−_.

### Characterizations of optical polarization evolution

For optical characterizations, a 50 × objective lens was used to focus the excitation laser beam on the tube wall, while the emitted photons were collected by the same objective and sent to the spectrometer. The polarization states of the resonant light were examined by a fixed polarizer in front of the detector of the spectrometer and a rotatable *λ*/2 plate. By rotating the *λ*/2 plate, the polarization orientation of the measured light can be rotated step-by-step and subsequently filtered by the polarizer and recorded by the detector. In this way, both the polarization orientation (with respect to the tube axis) and the polarization eccentricity can be resolved.

**Figure 2:**
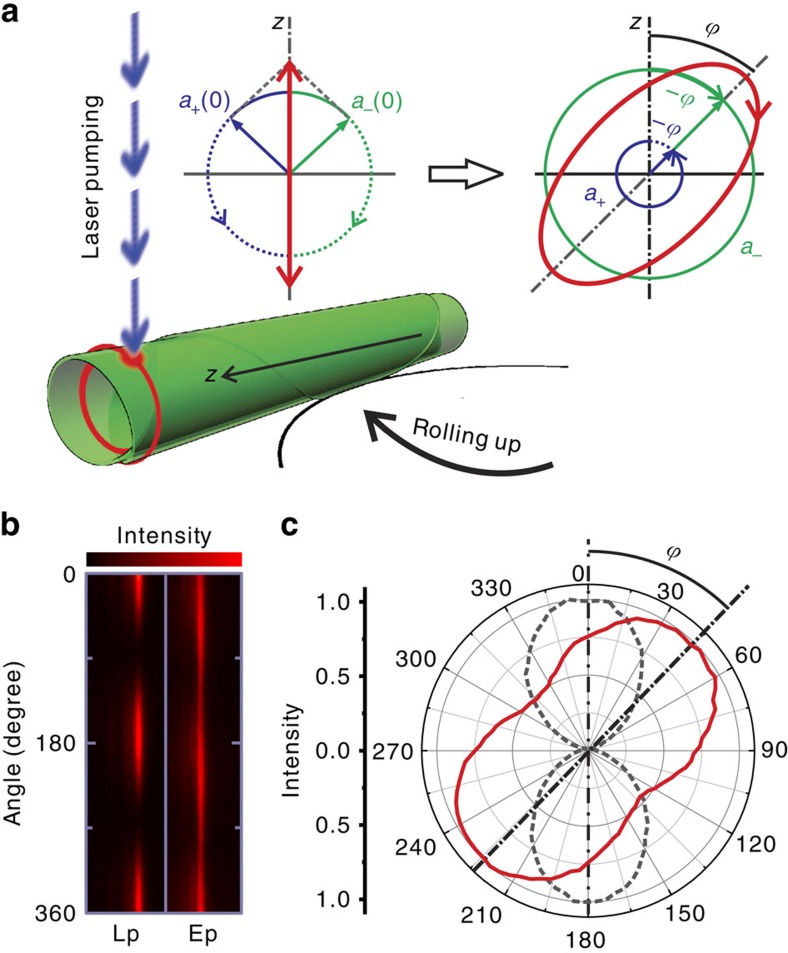
Elliptical polarization state of light in a cone-shaped microtube cavity. (**a**) In a rolled-up asymmetric microtube being pumped by a laser beam (532 nm), the linearly polarized light evolves into elliptically polarized one with the major axis tilted out of (with an angle *ϕ*) the tube axis. (**b**) Resonant mode intensity maps of a linear polarization (Lp) state measured from a symmetric tube where spin–orbit interaction is absent and an elliptical polarization (Ep) state measured in the presence of spin–orbit coupling of light in an asymmetric tube. In the corresponding polar diagrams shown in (**c**) the linear polarization (dashed line) is oriented parallel to tube axis while the elliptical polarization exhibits a tilt angle *ϕ* with respect to the tube axis.

It is well known that the resonant light in WGM microcavities is either transverse magnetic or transverse electric linearly polarized^22^. For symmetric microtubes, the measured electric field of the light is linearly polarized and oriented parallel to the tube axis for the transverse magnetic modes^22^. However, in cone-shaped microtube cavities the resonant light is no longer linearly polarized. Figure 2b shows the intensity maps for the linearly (Lp) and elliptically polarized (Ep) modes as a function of the orientation angle (0–360°), which were respectively measured from a symmetric and an asymmetric tube. In the intensity map measured from the symmetric tube, the polarization state is clearly shown to be linearly polarized along the tube axis. In the asymmetric tube case, the varying but unbroken polarization trace is characteristic for elliptical polarization. Moreover, the major axis of the ellipse, or in other words the polarization orientation, is found to tilt away from the tube axis. The polar plots in Fig. 2c clearly reveal the eccentricity and the tilt angle (*ϕ*∼44.5°) of one of the measured polarization states after evolution in the asymmetric microtube cavity. These unusual phenomena go beyond the conventional knowledge of optical WGM resonances in microcavities and can be attributed to the occurrence of a geometric phase in a non-Abelian evolution of light.

As mentioned above, the initial state of the resonant light in the microtube cavity is linearly polarized. A linear polarization state is comprised of the in-phase components of the right and left circular polarization components as **a**(0)=**a**_+_(0)+**a**_−_(0), with the same probability amplitude 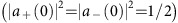, as schematically shown in Fig. 2a. Due to the spin–orbit coupling, the right and left circular components acquire a geometric phase with opposite signs: 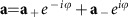, where *ϕ* is a geometric phase^3^, 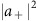 and 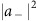 are redistributed vector amplitudes for each component due to the mode conversion, as described in equation (7). As shown in Fig. 2a, the conversion of amplitudes between the two circular components leads to a change from a linear to an elliptical polarization, while the geometric phase causes the orientation of the major axis of the polarization to tilt by an angle (equal to *ϕ*) with respect to the initial orientation. Since the final output state differs from the initial one, the evolution generates a non-cyclic geometric phase. Here we show that the non-cyclic geometric phase can be readily measured by simply recording the tilt angle of the light polarization ellipse. The change of the circular bases is evidence for the lack of independent modes, which is a consequence of the intricate non-Abelian evolution as described above. Since the photons are guided in the tube wall and their polarization states vary smoothly, the evolution can be described by an adiabatic process^2^,^3^.

**Figure 3:**
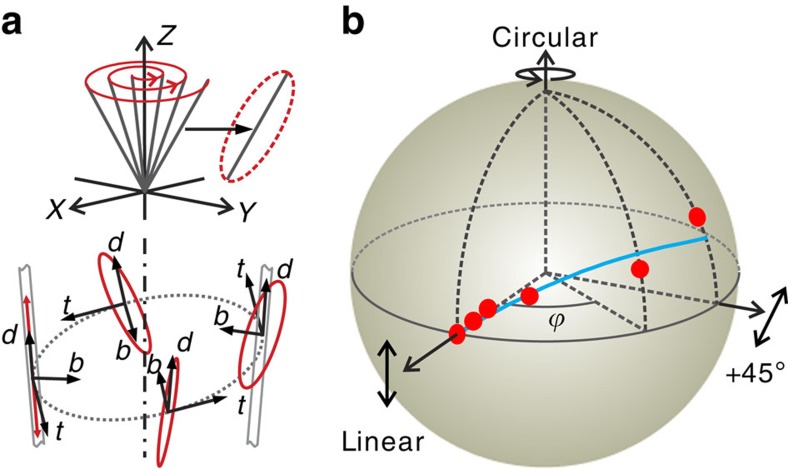
Non-cyclic evolution of degenerate photon systems in asymmetric microcavities. (**a**) The polarization state of light circulating in an asymmetric tube changes from linear to elliptical, which is visualized by defining a wave-accompanying coordinate (*t, d, b*) frame (bottom panel). In this process, the major axes of the evolving polarization state assumes a spiral around the tube axis which is shown with respect to the laboratory coordinate frame (top panel). (**b**) A series of measured polarization states (red dots) in the asymmetric tubes are plotted on a Poincaré sphere, which are fitted (blue curve) based on equation (6).

The resonant light experiences the spin–orbit coupling in an anisotropic medium when resonating in an asymmetric microtube cavity, hence the polarization state (described by the eccentricity and the tilt angle) continuously changes as the light resonates in the microtube, as schematically shown in Fig. 3a. However, the polarization state can only be measured when the light escapes from the microtube cavity, at which point the final state of the evolution has been reached. In order to depict the evolution trace, a series of final polarization states were measured from different asymmetric tubes, in which the resonant light experiences different extents of the polarization evolution. Figure 3b shows these series of polarization states plotted on a Poincaré sphere. In our measurements, tilt angles (Berry phase) up to ∼44.5° and an eccentricity of 0.7 have been recorded. It is found that a larger eccentricity is accompanied by a larger tilt angle (*ϕ*) due to their coupled evolution in equation (3). The corresponding evolution trace can be well reproduced by equation (6), indicating a good agreement between the theoretical model and measurements (see Supplementary Note 6). In addition, we have performed polarization measurements for different mode frequencies in the same tube cavity and found that the tilt angle as well as the eccentricity is independent of the wavelength. This is a clear evidence that the effect is of purely geometric, rather than dynamical origin.

**Figure 4:**
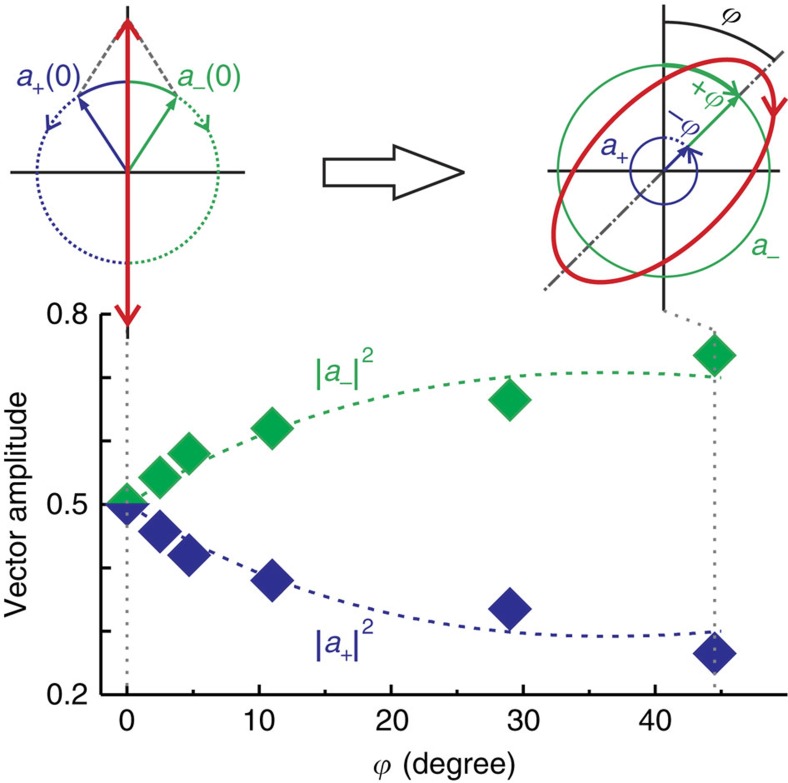
Change in the magnitude of the polarization components. Measured vector amplitudes of the right (**a**_+_) and left (**a**_−_) components with the concurrent geometric phase *ϕ*. The evolution traces agree well with the theoretical model in equation (7) (dashed curves). Top panel shows (left) a linear polarization comprised of in-phase rotating right and left circular polarization components, and (right) geometric phase +*ϕ* (shown with bold green arc) acquired for **a**_−_ and −*ϕ* (shown with dotted blue arc) acquired for **a**_−_.

In contrast to previous reports on optical spin–orbit coupling^3^,^7^,^8^, where the right and left handed circular polarization bases are often spatially separated, here we do not observe such a spatial separation of the spin components, but rather an amplitude conversion between basis vectors during the evolution, as discussed above. This process is systematically shown in Fig. 4 by comparing the variation of the squared moduli of the coefficients 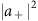 and 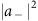 accompanied by the tilt angle *ϕ*. In the measured elliptical polarization curves, the maximum intensity represents the sum of the two moduli squared 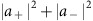, while the minimum represents the difference 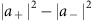. Based on the measured results, the respective squared amplitudes for the right 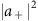 and left 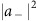 circular components are extracted. The two squared vector amplitudes vary in an opposite way and therefore result in the vector splitting of the spinning photons in a Hilbert space. The evolution traces of the two vector amplitudes agree well with the theoretical model of equation (7), as shown in Fig. 4.

## Discussion

In a previous report, light propagating around a dielectric microsphere cavity was used to mimic the effect of gravitational lensing^35^. Furthermore, the analogy between a static gravitational field and an anisotropic medium has been utilized to realize a spin–Hall effect triggered by gravitational field^36^. In this sense, our asymmetric microtube cavity could provide an effective analogue for the laboratory study of the light evolution in a gravitational field. Moreover, in WGM microcavities light is confined in a small volume. This avoids a large space required in the previously reported open light-path systems^3^,^29^, and is therefore attractive for integrating photonic applications on a chip. This finding may motivate the search for many novel applications, such as those for on-chip quantum information technologies, or exploiting interactions of light with chiral molecules^37^.

Our work shows that the non-cyclic geometric phase and the mode conversion for degenerate photon systems, in a non-Abelian evolution, can be readily demonstrated in a compact optical microtube cavity. The cone-like asymmetric optical microcavities establish an ideal platform to realize spin–orbit coupling for the examination of non-trivial topological effects in the context of a non-Abelian evolution. In our microtube structures, the geometric phase can be directly measured by simply monitoring the polarization tilt angles, while the eccentricities indicate the mode conversion between the right and left circular bases. Geometric phase and amplitude variations of components in the circular polarization basis reveal essential physical processes in a non-Abelian evolution, which is of interest for both fundamental and applied physics.

## Methods

### Microtube preparation

In our experiment, tubular microcavities were prepared by rolling-up pre-strained nanomembranes^30^,^38^,^39^. The cone-like microtubes were self-assembled by curling up a circularly patterned SiO_*x*_/SiO_2_ bilayer nanomembrane on a silicon substrate^30^, forming an asymmetric microtube. After roll-up, a 30-nm-thick hafnium oxide film was grown on the microtube surface using atomic-layer-deposition. The tube is around 7 μm in diameter with a wall thickness of about 100 nm. The tube length is 45 μm and the polarization states were measured at the larger-diameter-end, where high-*Q* resonant modes exist^30^. All high-*Q* resonant modes in an asymmetric tube exhibit the same output polarization state.

### Optical measurements

The optical polarization analyzer consists of a fixed polarization beam splitter in front of a spectrometer detector and an assisted rotatable half wave plate. The microtubular cavities are measured using a laser confocal microscope (50 × ), by which the excitation laser beam (at 532 nm) is focused at an area of 1 μm^2^ on the tube wall. The emission signal is collected through the same objective and then sent to the polarization analyzer. The initial orientation of the polarization beam splitter and half wave plate is calibrated by a predefined linearly polarized light, where the polarization orientation is set parallel to the tube axis. In the measurements, the eccentricities as well as the major axis orientation of the emission light polarizations are revealed by rotating the half wave plate (in a step of 2°).

## Additional information

**How to cite this article:** Ma, L. B. *et al*. Spin–orbit coupling of light in asymmetric microcavities. *Nat. Commun.* 7:10983 doi: 10.1038/ncomms10983 (2016).

## Supplementary Material

Supplementary Information Supplementary Figures 1-8, Supplementary Notes 1-6 Supplementary References.
