# Supplementary material for: Spin–orbit coupling of light in asymmetric microcavities
Source: Nat Commun. 2016 Mar 18;7:10983. doi: 10.1038/ncomms10983 (PMC4802038; doi:10.1038/ncomms10983)
Supplement: Supplementary Information — Supplementary Figures 1-8, Supplementary Notes 1-6 Supplementary References. [file ncomms10983-s1.pdf]

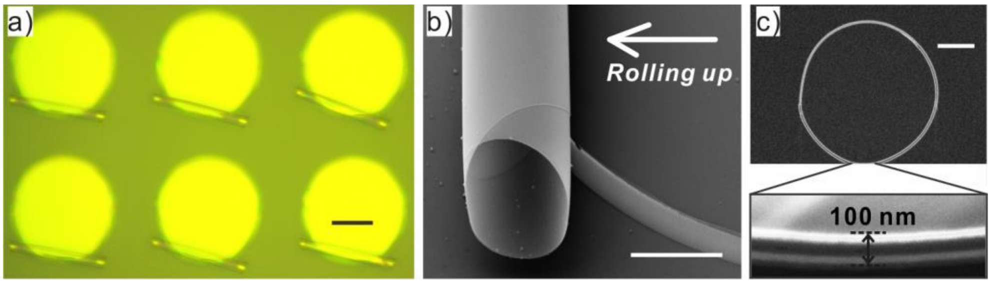


**Supplementary Figure 1**. **Images of rolled-up microtubes**. a) Optical image of asymmetric microtubes rolled up from circular patterns. Scale bar, 20 μm. b) A SEM image of a microtube prepared by rolling up a nanomembrane. Scale bar, 5 μm. c) Cross-sectional SEM image (Scale bar, 2 μm.) of a rolled-up microtube revealing multi-layered thin tube wall structure (see bottom panel), where the $HfO_2$ layers appear bright and the middle $SiO_x/SiO_2$ nanomembrane appears dark.

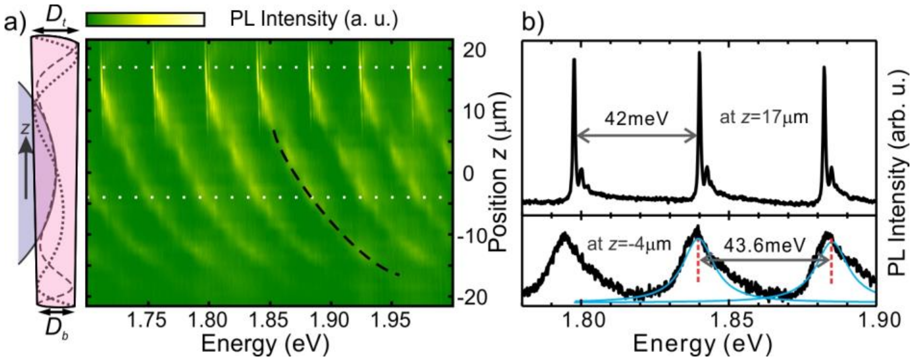


**Supplementary Figure 2**. **Optical characterizations of the asymmetric shape of microtube**. (a) Color-coded photoluminescence intensity map along the axis of an asymmetric tube. The left panel shows a sketch of the microtube cavity. (b) In the top panel, a spectrum showing three groups of high-*Q* resonant modes. In the bottom panel, a spectrum showing three groups of low-Q resonant modes. The blueshift of azimuthal modes and the decrease of mode spacing imply the tube diameter variation.

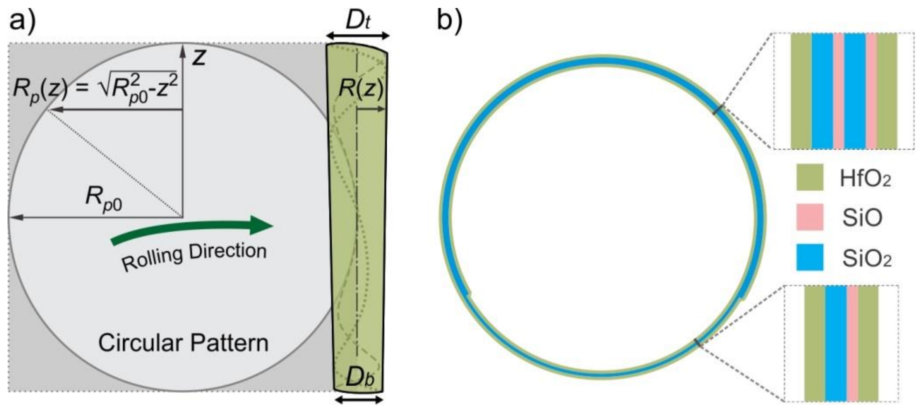


**Supplementary Figure 3**. **Geometry of rolled-up microtube**. (a) Schematic top view of an asymmetric tube rolled-up from a circular nanomembrane and (b) sketch of the spiral cross section of the rolled-up tube showing a layer structure of the tube wall.

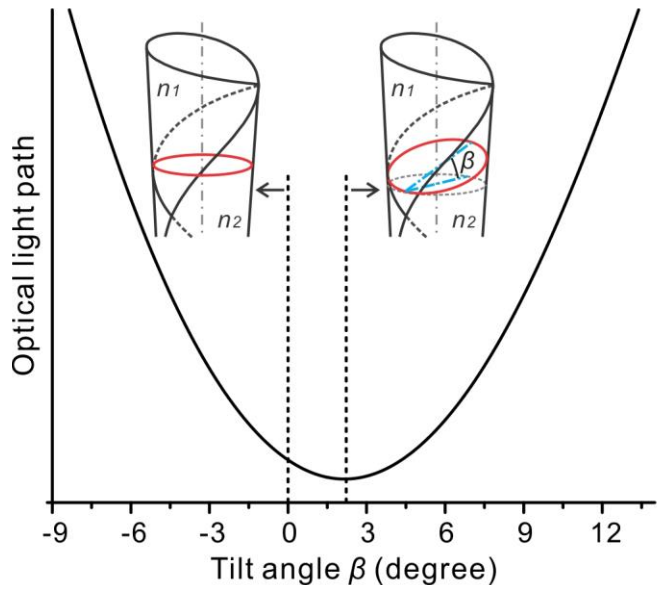


**Supplementary Figure 4**. **Calculation of the tilted optical trajectory in asymmetric microtube**. The optical light path reaches a minimum on an inclined trajectory due to the inhomogeneity of the tube wall. The tilt angle at the minimum of optical path depends on the tube geometry.

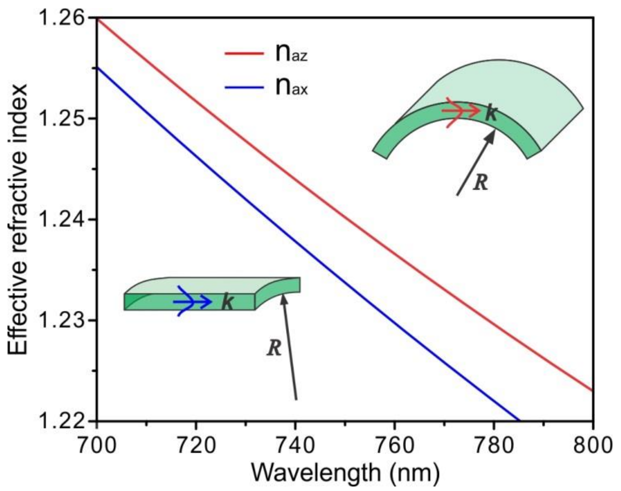


**Supplementary Figure 5**. **Anisotropy of the tube cavity**. Effective refractive indices of $n_{ax}$ and $n_{az}$ are plotted as a function of wavelength, manifesting an anisotropic nature in the microtubular structure.

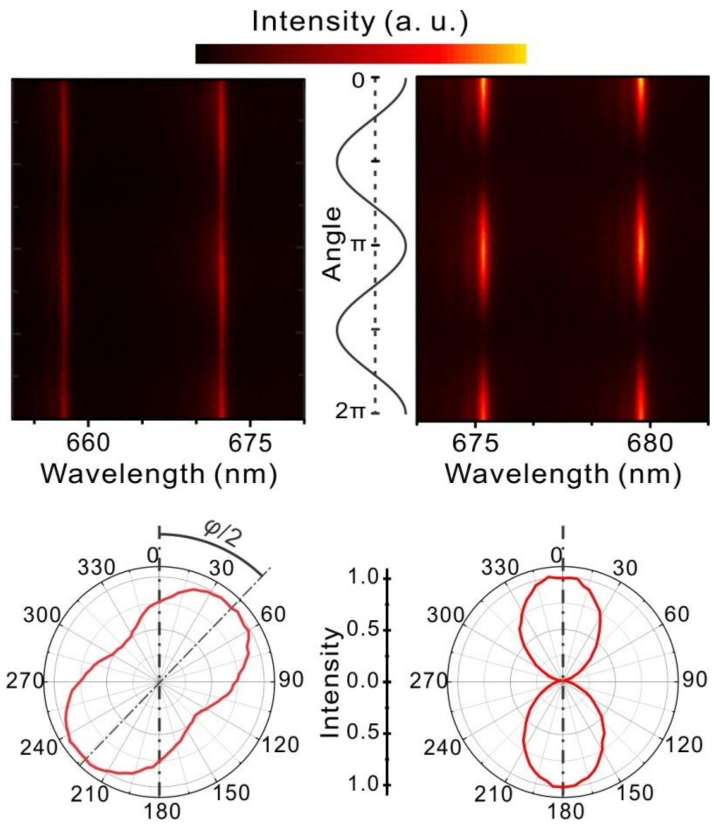


**Supplementary Figure 6**. **Characterization of polarization states**. A comparison between elliptical (left panel) and linear polarization (right panel) measured in the asymmetric and

symmetric tubes, respectively. The top panel is a PL intensity map, and the bottom panel shows the corresponding polarization state plotted in a polar diagram.

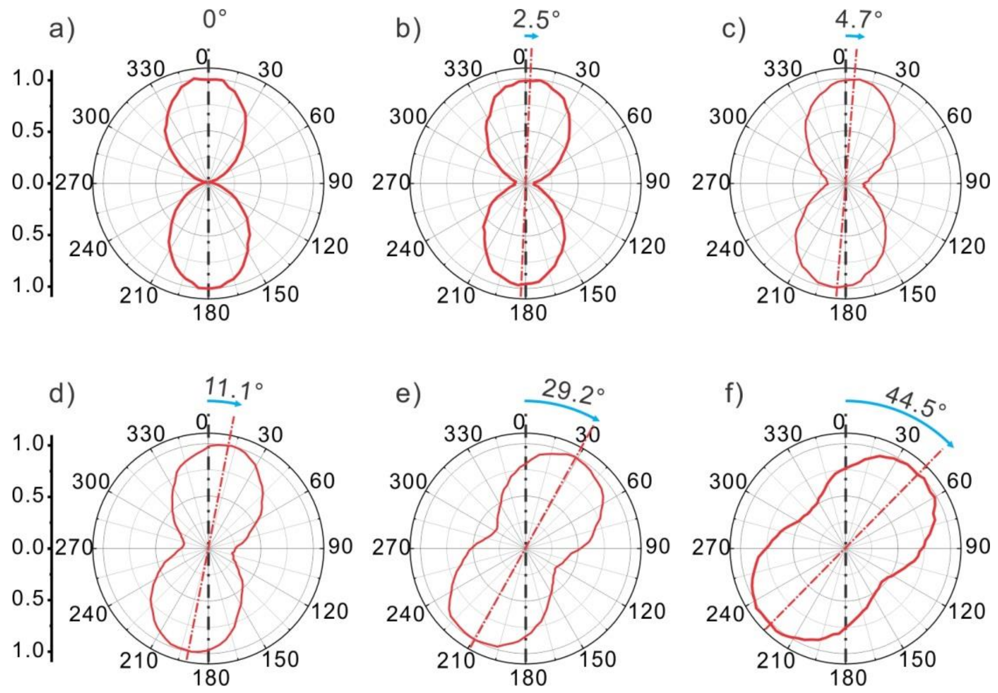


**Supplementary Figure 7**. **Measured polarization states**. The polarization states evolve from linear to elliptical polarization states with increasing the tilt angles and the ellipticities (panels from a to f). These polarization states were plotted on a Poincaré sphere as shown in Fig. 3. The ellipticity variations indicate a mode conversion between right and left polarization components, which were shown in Fig. 4.

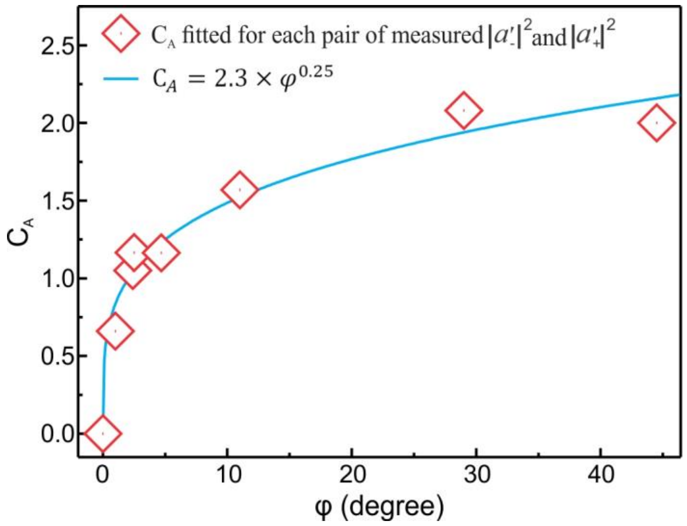

**Supplementary Figure 8**. **Relation between $C_A$ and φ**. $C_A$ values (red diamond) obtained by fitting the pair of measured $|a_+|^2$ and $|a_-|^2$ at each phase $\varphi$. The comparison between $C_A$ and $\varphi$ in our sample implies a relation $C_A = 2.3 \times \varphi^{0.25}$, which is used to represent the overall evolution traces shown in Figs. 3 and 4. To better justify the relation between $C_A$ and $\varphi$, two additionally measured at 1.0 °and 2.4 °positions data were added.

**Supplementary Note 1 Rolled-up asymmetric microtube cavities**

Microtube cavities were fabricated by releasing differentially strained $SiO_x/SiO_2$ bilayer nanomembranes (in circular pattern) which curl into microtube structures on a silicon substrate. In the present work, cone-like asymmetric microtubes were prepared by releasing and rolling up tubes in an uneven fashion as discussed previously [see Ref. (*29*)]. The resonators were subsequently coated with a layer of $HfO_2$ by atomic layer deposition (ALD) to modify their effective refractive indices. As shown in Supplementary Fig. 1, the tube has less than two windings in the end of the tube, where the measurements were performed. The layered thin tube wall (~100 nm) was examined using cross-sectional scanning electron microscopy (SEM). The white traces in outer sides of the tube wall correspond to the $HfO_2$ layer, while the middle dark layer corresponds to the $SiO_x/SiO_2$ nanomembrane. The refractive index of the tube wall is calculated by averaging the refractive indices of each layer in the tube wall. Therefore, the averaged refractive index of the tube wall varies along the tube axis due to the variation of the number of windings.

**Supplementary Note 2 Conical angle estimation**

Optical resonant modes can directly reflect the tube diameter based on azimuthal resonant condition $\pi D = M\lambda_M/n_{eff}$, where $D$ is the tube diameter, $M$ is azimuthal mode number, $\lambda_M$ is resonant wavelength, and $n_{eff}$ is effective refractive index. Photoluminescence intensity mapping was performed by scanning along the tube axis, as shown in Supplementary Fig. 2. The resonant modes of high quality ($Q$) factor are located at the larger diameter side of the

tube due to the enhanced light confinement. The lower-$Q$ azimuthal modes continuously blueshift, while scanning from the large to small diameter region. The continuous blueshift of the resonant modes is a direct evidence of the diameter variation $\Delta D$ along the tube axis, which can be calculated by considering the azimuthal resonant condition $\pi D = M\lambda_M/n_{eff}$. The microtube has a mean diameter of 7 μm and a diameter difference $\Delta D$ (= $D_t$ - $D_b$) of ~ 1.5 μm between the diameters $D_t$ at the top end and $D_b$ at the bottom end. Based on these measurements, the conical angle of the asymmetric tube was estimated to be ~2 °.

**Supplementary Note 3 Inclined trajectory calculation**

For the optical light path calculation, a geometric model of the asymmetric tube cavity was built up by defining the size of the initial circular pattern as well as the parameters of rolling.

In the larger diameter side of the tube, the variation of windings along the tube axis results in distinct thick and thin parts (with thickness $T_1$ and $T_2$, respectively). The averaged refractive indices are represented by $n_1$ and $n_2$ for the thin and thick parts, respectively. The lengths of the spiral cross sections are obtained by integrating the middle radius ($R_{int}$+$T_i$/2, i = 1, 2) of the thick part (with length $L_1$) and thin part (with length $L_2$) of the spiral tube wall. They are

$$L_1 = \int_{\theta=0}^{\theta 1}\left(R_{\text{int}}+\frac{T_1}{2}\right)d\theta = \left(R_0+\frac{T_1}{2}\right)\theta_1+\frac{T\theta_1^2}{4\pi} \tag{1}$$

and

$$L_2 = \int_{\theta 1}^{2\pi}\left(R_{\text{int}}+\frac{T_2}{2}\right)d\theta \,\Big|_{\theta 1=2\pi-\theta_2} = \left(R_0+\frac{T_2}{2}\right)\theta_2+T\theta_2-\frac{T\theta_2^2}{4\pi} \tag{2}$$

The angle $\theta_1$ is determined by $\theta_1 = 2\pi p_1$ (and $\theta_2=2\pi-\theta_1$), where $p_1 = L_1/(L_1+L_2)$ and $p_2 = 1-p_1$. Since the $\theta_i$ depends on the $L_i$, Eqs. (1) and (2) must be solved in self-consistent way.

The thick and thin parts are considered separately as follows: We first estimate the $n_{\text{avg}}^{(i)}, i = 1,2$ by averaging the refractive index along the multilayer wall structure. The following formula is used:

$$n_{\text{avg}}^{(i)} = \left[\sum_{j=1}^{N_i} T_j n_j^2 \Big/ \left(\sum_{j=1}^{N_i} T_j\right)\right]^{1/2}, \tag{3}$$

where $T_j$ is the thickness of the layer $j$ on the tube wall (with the refractive index $n_j$) , and $N_i$ is the number of layers in the thick or thin parts, respectively. Each subscript refers to an individual layer in the multilayered tube wall. In this way each multilayer wall structure is approximated by a single layer planar waveguide and the resulting averaged, effective refractive index[1] is used in the following calculations.

The optical paths were calculated as a function of the inclination angles. At each inclination angle, the optical path is separated into two parts, $L_1(\beta)$ and $L_2(\beta)$ located in thin and thick part with effective refractive indices of $n_{\text{avg}}^{(1)}$ and $n_{\text{avg}}^{(2)}$, respectively. The total light path is $F = n_{\text{avg}}^{(1)} L_1(\beta) + n_{\text{avg}}^{(2)} L_2(\beta)$. The $L_1(\beta)$ and $L_2(\beta)$ are obtained by numerical integration using MATLAB. The shortest optical path is obtained if the trajectory inclined by a few degrees. This result indicates the light should travel along a slightly inclined trajectory in accordance with Fermat's principle.

**Supplementary Note 4 Anisotropic refractive indices in the microtube cavity**

The effective refractive index is calculated by solving Maxwell's equations in the microtubular curved structure[1]. In a planar slab waveguide, a first-order approximation is sufficient for the calculation of the effective refractive index. In the microtubular curved structure, the effect of curvature should be considered, and therefore, a second-order correction needs to be taken into account.

For the case of light propagating along the tube axis, the effective refractive index $n_{\text{avg}}^{(x)}$ is calculated by solving the equation

$$\left[\frac{\partial^2}{\partial\rho^2} + \frac{1}{\rho}\frac{\delta}{\partial\rho} - \frac{1}{\rho^2} + \varepsilon(\rho)\left(\frac{\omega}{c}\right)^2\right]F = \varepsilon_{\text{avg}}^{(x)}\left(\frac{\omega}{c}\right)^2 F. \quad (4)$$

Similarly, for the case of light propagating in the azimuthal direction, the effective refractive index $n_{\text{avg}}^{(\theta)}$ is calculated using the equation

$$\left[\rho^2\frac{\partial^2}{\partial\rho^2} + \rho\frac{\delta}{\partial\rho} - \frac{1}{\rho^2} + \rho^2\varepsilon(\rho)\left(\frac{\omega}{c}\right)^2\right]F = \varepsilon_{\text{avg}}^{(\theta)}\left(\frac{\omega}{c}\right)^2 F. \quad (5)$$

In Supplementary Fig. 5, the effective refractive indices of $n_{\text{avg}}^{(x)}$ and $n_{\text{avg}}^{(\theta)}$ are plotted as a function of wavelength. It is shown that the microtube cavity exhibits an anisotropic effective

refractive index. Along the inclined trajectory imposed by Fermat's principle, the light will experience a weakly anisotropic and inhomogeneous medium, satisfying the condition for a non-Abelian evolution as theoretically discussed in Ref. (*17*).

**Supplementary Note 5 Measurement results of polarization states**

Polarization state of the emitted light was examined with the measurement setup shown in Fig. 2. Linear polarization states were found in symmetric microtube cavities, with the polarization oriented parallel to tube axis, as shown in Supplementary Fig. 6. In contrast to symmetric microtubes, elliptical polarization states were revealed in asymmetric microcavities, where the polarization orientation was greatly biased away from the tube axis.

**Supplementary Note 6 Derivation of the theoretical model to fit measurements**

The mapping matrix in Eq. (6) in the manuscript reads

$$\widehat{\mathrm{M}} = \exp\begin{pmatrix} -i\varphi & iC_{\mathrm{A}} \\ iC_{\mathrm{A}} & i\varphi \end{pmatrix} = \exp[i(-\varphi\widehat{\sigma}_3 + C_{\mathrm{A}}\widehat{\sigma}_1)], \quad (6)$$

which can be expanded in Taylor series as

$$\widehat{\mathrm{M}} = \hat{\mathrm{I}} + i(-\varphi\widehat{\sigma}_3 + C_{\mathrm{A}}\widehat{\sigma}_1) + \frac{i^2}{2!}(-\varphi\widehat{\sigma}_3 + C_{\mathrm{A}}\widehat{\sigma}_1)^2 + \frac{i^3}{3!}(-\varphi\widehat{\sigma}_3 + C_{\mathrm{A}}\widehat{\sigma}_1)^3 + \frac{i^4}{4!}(-\varphi\widehat{\sigma}_3 + C_{\mathrm{A}}\widehat{\sigma}_1)^4 + \cdots \quad (7)$$

where $\hat{\mathrm{I}}$ is a unit matrix. Taking into account the basic properties of the Pauli matrices[2]: ${\widehat{\sigma}_1}^2 = {\widehat{\sigma}_3}^2 = \hat{\mathrm{I}}$; $\widehat{\sigma}_3\widehat{\sigma}_1 + \widehat{\sigma}_1\widehat{\sigma}_3 = 0$, Equation (7) reads

$$\widehat{\mathrm{M}} = \hat{\mathrm{I}} + i(-\varphi\widehat{\sigma}_3 + C_{\mathrm{A}}\widehat{\sigma}_1) + \frac{i^2}{2!}\left(\varphi^2 + {C_{\mathrm{A}}}^2\right)\hat{\mathrm{I}} + \frac{i^3}{3!}(-\varphi\widehat{\sigma}_3 + C_{\mathrm{A}}\widehat{\sigma}_1)\left(\varphi^2 + {C_{\mathrm{A}}}^2\right) + \frac{i^4}{4!}\left(\varphi^2 + {C_{\mathrm{A}}}^2\right)^2\hat{\mathrm{I}} + \cdots \quad (8)$$

Arranging coefficients for each of the independent matrices, a compact expression for the mapping matrix is found

$$\widehat{\mathrm{M}} = \hat{\mathrm{I}}cos\sqrt{\varphi^2 + {C_{\mathrm{A}}}^2} + i(-\varphi\widehat{\sigma}_3 + C_{\mathrm{A}}\widehat{\sigma}_1)\frac{sin\sqrt{\varphi^2 + {C_{\mathrm{A}}}^2}}{\sqrt{\varphi^2 + {C_{\mathrm{A}}}^2}}$$

$$= \begin{pmatrix} \cos\sqrt{\varphi^2 + C_\mathrm{A}{}^2} - i\varphi \frac{\sin\sqrt{\varphi^2+C_\mathrm{A}{}^2}}{\sqrt{\varphi^2+C_\mathrm{A}{}^2}} & iC_\mathrm{A} \frac{\sin\sqrt{\varphi^2+C_\mathrm{A}{}^2}}{\sqrt{\varphi^2+C_\mathrm{A}{}^2}} \\ iC_\mathrm{A} \frac{\sin\sqrt{\varphi^2+C_\mathrm{A}{}^2}}{\sqrt{\varphi^2+C_\mathrm{A}{}^2}} & \cos\sqrt{\varphi^2 + C_\mathrm{A}{}^2} + i\varphi \frac{\sin\sqrt{\varphi^2+C_\mathrm{A}{}^2}}{\sqrt{\varphi^2+C_\mathrm{A}{}^2}} \end{pmatrix}. \qquad (9)$$

Taking Supplementary Eq. (9) to Eq. (6) in the manuscript, the final state of light is described as

$$\begin{pmatrix} a_+ \\ a_- \end{pmatrix} = \widehat{\mathrm{M}} \frac{1}{\sqrt{2}} \begin{pmatrix} 1 \\ 1 \end{pmatrix} = \frac{1}{\sqrt{2}} \begin{pmatrix} \cos\sqrt{\varphi^2 + C_\mathrm{A}{}^2} + i(\varphi - C_\mathrm{A}) \frac{\sin\sqrt{\varphi^2+C_\mathrm{A}{}^2}}{\sqrt{\varphi^2+C_\mathrm{A}{}^2}} \\ \cos\sqrt{\varphi^2 + C_\mathrm{A}{}^2} + i(\varphi + C_\mathrm{A}) \frac{\sin\sqrt{\varphi^2+C_\mathrm{A}{}^2}}{\sqrt{\varphi^2+C_\mathrm{A}{}^2}} \end{pmatrix} \qquad (10)$$

where

$$|a_+|^2 = \frac{1}{2}\left( 1 - 2\varphi C_\mathrm{A} \frac{\sin^2\sqrt{\varphi^2+C_\mathrm{A}{}^2}}{\varphi^2+C_\mathrm{A}{}^2} \right); \ |a_-|^2 = \frac{1}{2}\left( 1 + 2\varphi C_\mathrm{A} \frac{\sin^2\sqrt{\varphi^2+C_\mathrm{A}{}^2}}{\varphi^2+C_\mathrm{A}{}^2} \right). \qquad (11)$$

The measured data shown in Fig. 4 were fitted one-by-one at each phase φ using Supplementary Eq. (11), as shown in Supplementary Fig. 8.

Based on the above analysis, the data shown in the Fig. 3 and 4 in the manuscript can be well fitted based on Supplementary Eq. (10) and (11) [i.e. Eq. (6) and (7) in the manuscript].

**Supplementary References:**


1. P. Yeh, *Optical Waves in Layered Media*; Wiley Interscience; New Jersey, 2005; Ch. 11.

2. L. D. Landau and E. M. Lifshitz, *Quantum mechanics: Non-relativistic theory* ($4^{th}$ edition); Moscow, Nauka, 1989; p. 249.
